# Energy Cost of Dynamical Stabilization: Stored versus Dissipated Energy

**DOI:** 10.3390/e24081020

**Published:** 2022-07-24

**Authors:** Armen E. Allahverdyan, Edvard A. Khalafyan

**Affiliations:** 1 Alikhanian National Laboratory, Yerevan Physics Institute, 2 Alikhanian Brothers Street, Yerevan 0036, Armenia; 2 Cosmology Center, Yerevan State University, 1 A. Manoogian Street, Yerevan 0025, Armenia; 3 Department of Applied Mathematics and Informatics, Moscow Institute of Physics and Technology, State University, 141701 Dolgoprudny, Moscow Oblast, Russia; khalafyan.ea@phystech.edu

**Keywords:** dynamical stabilization, Kapitza’s pendulum, energy storage, asymptotic stability

## Abstract

Dynamical stabilization processes (homeostasis) are ubiquitous in nature, but the needed energetic resources for their existence have not been studied systematically. Here, we undertake such a study using the famous model of Kapitza’s pendulum, which has attracted attention in the context of classical and quantum control. This model is generalized and rendered autonomous, and we show that friction and stored energy stabilize the upper (normally unstable) state of the pendulum. The upper state can be rendered asymptotically stable, yet it does not cost any constant dissipation of energy, and only a transient energy dissipation is needed. Asymptotic stability under a single perturbation does not imply stability with respect to multiple perturbations. For a range of pendulum–controller interactions, there is also a regime where constant energy dissipation is needed for stabilization. Several mechanisms are studied for the decay of dynamically stabilized states.

## 1. Introduction

Dynamical stabilization is an important concept in physics (particle trapping, Floquet engineering) [1,2,3,4], control theory (vibrational stabilization and robotics) [5,6,7,8], biology (homeostasis) [9,10,11,12], animal locomotion [13,14], and population dynamics (polymorphism in time-dependent environments) [15,16]. The meaning of this concept is that certain relevant parameters (concentrations, coordinates) are stabilized against external perturbations by active and frequently self-regulating means. This is achieved via specific engines or controllers, and no stability exists without their action.

Is there an energy cost for dynamic stabilization, and how is it estimated? This question is obviously relevant to controlling methods. A general explanation for homeostasis in biology is that it offers energetically cheaper realizations of physiological functions [9], which leads to asking about its own energy costs.

In order to study the energy cost problem, we chose a simple but nontrivial model that exhibits dynamical stabilization. This is the driven nonlinear pendulum whose upper (normally unstable state) can be stabilized by a sufficiently fast external force thereby defying gravity. Such models were first studied by Stephenon [17], and then by Kapitza [18,19]; see [20] for a review. They still produce new physical results [21,22,23] and have interesting applications [1,2,3,4,5,6,7,8,13,14].

Our first step is to replace the external field with a controller degree of freedom in order to render the driven pendulum autonomous. This ensures finite energies and accounts of all relevant degrees of freedom; see Section 2. The autonomous pendulum predicts the two following scenarios for the dynamical stabilization of the unstable state. Within the first scenario, the state is asymptotically stable. There are two factors behind this strong notion of stabilization: the energy stored in the controller that ensures the needed effective potential, and the friction acting on the pendulum (obviously, friction is necessary for asymptotic stability). There are no permanent energy costs here, i.e., once the asymptotically stable state is reached, the interaction with the controller is automatically switched off. There is only a moderate transient dissipation of energy during relaxation. The interaction emerges online together with an external perturbation.

However, the notion of asymptotic stability is not sufficient for characterizing this scenario of dynamic stabilization. Contrary to passively stabilized systems, asymptotic stability does not guarantee stability under a sequence of well-separated perturbations acting within the attraction basin. For characterizing this more general notion of stability, we again need the concept of stored energy.

The second scenario predicted by the model is realized when the back-reaction from pendulum to controller is sizeable. Here, asymptotic stability is replaced by a metastable stabilization that has a finite (though possibly long) lifetime because the controller steadily dissipates the stored energy for supporting the metastable state. Once this energy is lower than a certain threshold, the metastable state suddenly decays with dissipating away all the energy. Therefore, dynamic stabilization within this scenario requires permanent energy costs.

Thus, the model provides conceptual tools and scenarios for addressing the energy cost problem in more general (especially biological) dynamic stabilization situations.

This paper is organized as follows. The generalized Kapitza’s model is formulated in Section 2 and is approximately solved in Section 3. We show that the asymptotic stability of the inverted pendulum is determined (among other factors) by the energy stored in the controller. This extends the stability criterion presented in [18,19,20]. In Section 4, we confirm the analytic bound and numerically work out two basic scenarios of dynamic stability. Section 4.2 discusses a new scenario of noise-induced metastability. Our results are summarized in Section 5, where we also discuss possible biological implications of our research.

## 2. Pendulum and Controller

**Figure 1:**
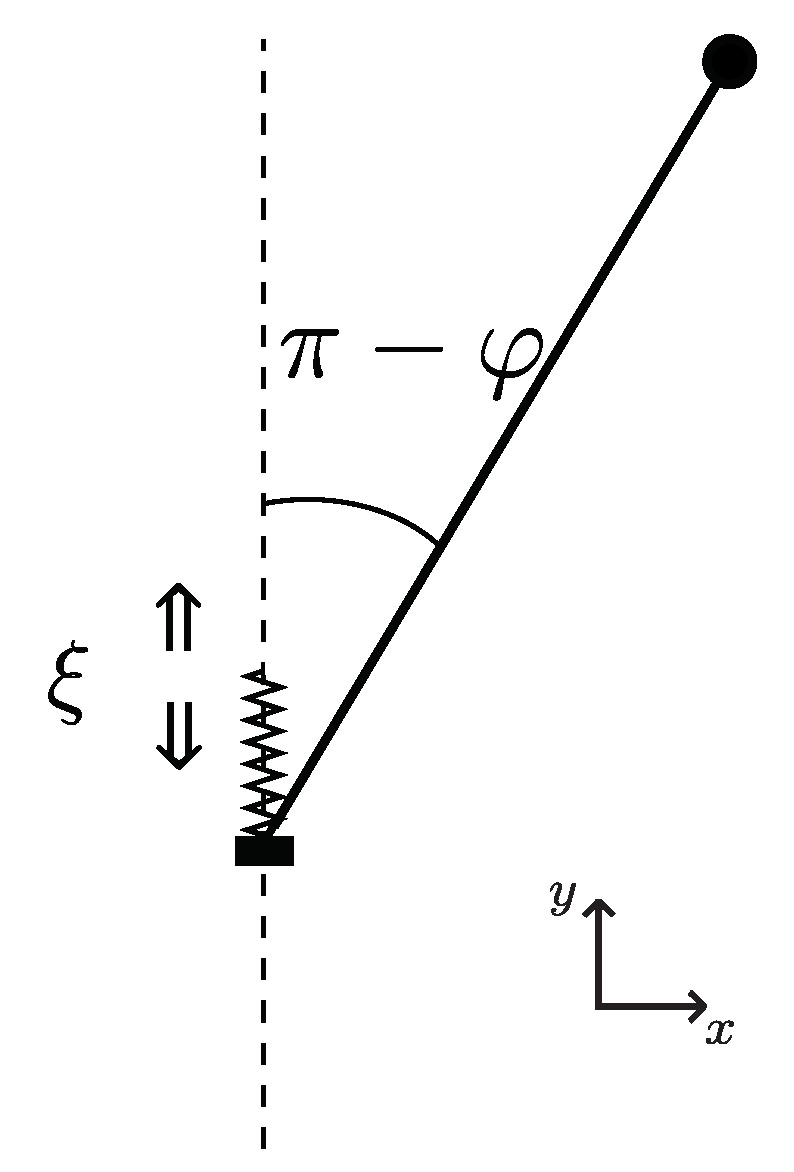
A schematic representation of the inverted pendulum. Angle φ is defined such that the upper (normally unstable) position refers to φ=π. The end point of the pendulum moves vertically with coordinate ξ that is subject to a harmonic potential; cf. (3).

As a result of its numerous applications, Kapitza’s pendulum can be introduced in a variety of contexts. For clarity, we introduce its generalization within a mechanical picture; cf. [19]. Consider a pendulum moving on (x,y) plane in a homogeneous gravity field *g*; see Figure 1. The pendulum is a material point with mass *m* fixed at one end of a rigid rod with length *l*. Let the coordinates and velocities of the mass be

(1) $$x=l\sin\varphi ,\;\dot{x}\equiv \frac{dx}{dt}=l\cos\varphi \cdot \dot{\varphi },$$

(2) $$y=\xi_{0}-l\cos\varphi ,\;\dot{y}\equiv \frac{dy}{dt}={\dot{\xi }}_{0}+l\sin\varphi \cdot \dot{\varphi },$$

where $\xi_{0}$ refers to the vertical (along *y* axes) motion of the opposite end of the road. The Lagrangian of the autonomous system with coordinates (x,y,ξ) reads

(3) $$L=\frac{m}{2}\left[{\dot{x}}^{2}+{\dot{y}}^{2}\right]-mgy+\frac{\mu }{2}{\dot{\xi }}_{0}^{2}-\frac{k}{2}\xi_{0}^{2},$$

where μ is the mass of $\xi_{0}$, and we assumed a harmonic potential $\frac{k}{2}\xi_{0}^{2}$ for $\xi_{0}$. Putting (1) and (2) into (3), denoting $\xi =\xi_{0}+\frac{mg}{k}$, and dropping a constant term from the Lagrangian, we find

(4) $$L=\frac{ml^{2}}{2}{\dot{\varphi }}^{2}+\frac{\mu +m}{2}{\dot{\xi }}^{2}+ml\dot{\xi }\dot{\varphi }\sin\varphi +mgl\cos\varphi -\frac{k}{2}\xi^{2},$$

which implies Lagrangian equations of motion $\frac{d}{dt}\frac{\partial L}{\partial \dot{q}}=\frac{\partial L}{\partial q}$ with coordinates q=(φ,ξ) and velocities $\dot{q}=\left(\dot{\varphi },\dot{\xi }\right)$:

(5) $$\ddot{\varphi }+\frac{g}{l}\sin\varphi =-\frac{1}{l}\sin\varphi \cdot \ddot{\xi },$$

(6) $$\ddot{\xi }+\omega^{2}\xi =-\epsilon \;\left[\;\cos\varphi \cdot {\dot{\varphi }}^{2}+\sin\varphi \cdot \ddot{\varphi }\;\right],$$

(7) $$\omega^{2}\equiv k/\left(\mu +m\right),\;\epsilon \equiv l/\left(1+\frac{\mu }{m}\right),$$

where ω is the frequency of ξ, while ϵ characterizes the back-reaction of *x* on ξ. From (3) and (6), whenever μ≫m, i.e., controller ξ is much heavier than the pendulum, we can neglect the left-hand side of (6) and revert to the usual (nonautonomous) driven pendulum. This, however, does not suffice for the full understanding of energy costs, which arise due to the very back-reaction of *x* on ξ.

Equations (5) and (6) are deduced from the time-independent Lagrangian (4); hence, they are conservative and reversible. The conserved energy related to (4) reads:

(8) $$E=\dot{\varphi }\frac{\partial L}{\partial \dot{\varphi }}+\dot{\xi }\frac{\partial L}{\partial \dot{\xi }}-L=\frac{ml^{2}}{2}{\dot{\varphi }}^{2}+\frac{\mu +m}{2}{\dot{\xi }}^{2}+ml\dot{\xi }\dot{\varphi }\sin\varphi -mgl\cos\varphi +\frac{k}{2}\xi^{2}.$$

We now add a friction with parameter γ>0 to (5), writing it as

(9) $$\ddot{\varphi }+\frac{g}{l}\sin\varphi +\gamma \dot{\varphi }=-\frac{1}{l}\sin\varphi \cdot \ddot{\xi }.$$

As seen below, this friction is a means of stabilizing the motion of φ. We did not add a friction to the controller degree of freedom ξ, since this achieves no constructive goal besides providing an additional channel for loosing energy. We also mostly neglect random noises acting on φ, i.e., we do not study Langevin equations. The influence of a random noise is discussed in Section 4.2.

Energy (8) governed by (6) and (9) decays in time, as it should:

(10) $$\frac{dE}{dt}=-ml^{2}\gamma {\dot{\varphi }}^{2}\leq 0.$$

As confirmed below, it is useful to separate energy *E* in (8) into two contributions, those describing the motion of ξ and φ:

(11) $$E=E_{\varphi }+E_{\xi },\;E_{\xi }=\frac{\mu +m}{2}{\dot{\xi }}^{2}+\frac{k}{2}\xi^{2}.$$

## 3. Solving the Model via Slow and Fast Variables

To solve nonlinear (6) and (9), we apply both the separation of time scales and perturbation theory. We assumed that ω in (6) is a large parameter, i.e., ξ oscillates fast. Next, we separate φ as [19]:

(12) φ=Φ+ζ,ζ≪Φ

where Φ is slow, and ζ is fast and also small compared with Φ. Then, from (6), (9), and (12), after expanding over ζ and keeping the first nonvanishing term only, we obtain:

(13) $$\ddot{\Phi }+\ddot{\zeta }+\frac{g}{l}\sin\Phi +\zeta \frac{g}{l}\cos\Phi +\gamma \dot{\Phi }+\gamma \dot{\zeta }=-\frac{1}{l}\sin\Phi \cdot \ddot{\xi }-\frac{1}{l}\cos\Phi \cdot \zeta \ddot{\xi },$$

(14) $$\ddot{\xi }+\omega^{2}\xi =-\epsilon \left[\left(\cos\Phi -\zeta \sin\Phi \right){\left(\dot{\Phi }+\dot{\zeta }\right)}^{2}+\left(\sin\Phi +\zeta \cos\Phi \right)\left(\ddot{\Phi }+\ddot{\zeta }\right)\right].$$

Assuming that $\gamma ≳\frac{1}{\omega }$, and noting that, for fast variables, $\dot{\zeta }$, $\ddot{\zeta }$, $\dot{\xi }$, and $\ddot{\xi }$ are large, we can equalize fast and large components in (13) and (14):

(15) $$\ddot{\zeta }+\gamma \;\dot{\zeta }=-\frac{1}{l}\sin\Phi \cdot \ddot{\xi },$$

(16) $$\ddot{\xi }+\omega^{2}\xi =-\epsilon \sin\Phi \cdot \ddot{\zeta },$$

(17) ζ(0)=0,

where initial condition (17) is imposed without loss of generality; cf. (12). In (16) we particularly neglected the factor $-\epsilon \cos\Phi \cdot {\dot{\zeta }}^{2}$, because it is quadratic over ζ. Equations (15) and (16) do not contain the contribution $\zeta \frac{g}{l}\cos\Phi$ coming from the potential −mglcosφ [cf. (13)], since this contribution is not sufficiently fast, i.e., (15) and (16) involve only time-derivatives of ζ.

Equation (15) can be integrated over the time. A constant of integration should be put to zero, since ζ and $\dot{\xi }$ are oscillating in time, with their time average being zero. Hence, the integration of (15) implies

(18) $$\dot{\zeta }\left(0\right)=-\frac{\sin\Phi }{l}\dot{\xi }\left(0\right).$$

Equations (15) and (16) are linear over unknown variables ζ and ψ, and they can be solved via the Laplacean transform (see Appendix A). Now, we can average (13) over fast oscillations:

(19) $$\ddot{\Phi }+\frac{g}{l}\sin\Phi +\gamma \dot{\Phi }+\frac{1}{l}\cos\Phi \cdot \bar{\zeta \left(t\right)\;\ddot{\xi }\left(t\right)}=0,$$

where $\bar{...}$ means time averaging over fast oscillations. Factor $\bar{\zeta \left(t\right)\;\ddot{\xi }\left(t\right)}$ in (19) is worked out in Appendix A. It contributes to an effective (generally time-dependent) potential Π(Φ):

(20) $$\ddot{\Phi }+\gamma \dot{\Phi }=-\partial_{\Phi }\Pi \left(\Phi \right).$$

The form of this potential is simplified if we assume that a slow variable $\beta \equiv \frac{\sin^{2}\Phi }{1+\frac{\mu }{m}}$ holds β≪1 due to $\frac{\mu }{m}\gg 1$ (weak back-reaction), and take the first nonvanishing term over β; see Appendix A. This approximation is only supported when slow variable Φ relaxes to π or to 0. Eventually, we have a simplified form of the effective potential:

(21) $$\Pi =-\frac{g}{l}\cos\Phi -\frac{\omega^{2}}{\gamma^{2}+\omega^{2}}\cdot \frac{{\dot{\xi }}^{2}\left(0\right)+\omega^{2}\xi^{2}\left(0\right)}{8l^{2}}\cdot \cos2\Phi .$$

Now, Φ=π is stable, i.e., $\partial_{\Phi }{\Pi \left(\Phi \right)|}_{\Phi =\pi }=0$ and $\partial_{\Phi }^{2}{\Pi \left(\Phi \right)|}_{\Phi =\pi }>0$, if:

(22) $$\frac{{\dot{\xi }}^{2}\left(0\right)+\omega^{2}\xi^{2}\left(0\right)}{2gl}>1+\frac{\gamma^{2}}{\omega^{2}}.$$

Larger values of ω expectedly increase the stability domain. However, larger values of friction constant γ decrease it. Hence, friction plays a double role in this system, since the very relaxation of Φ(t) (e.g., Φ(t)→π) is achieved due to friction. Note that the authors in [23] studied the inverted pendulum with friction and deduced an effective potential that is akin to (21) (and even contains higher-order terms), but does not contain friction explicitly since the latter was assumed to be small.

Equations (20) and (21) imply that, when Φ=π is a stable rest point, we obtain that the slow part Φ(t) of the angle variable φ(t) convergence due to the friction: Φ(t)→π, if Φ(0) is in the attraction basin of Φ=π. What happens to the fast part ζ(t) of φ(t)? This is a convoluted question that we numerically clarify below. Our results in Appendix A show that, when derivation (12)–(18) applies, i.e., both time-scale separation and the perturbation over β hold, we obtain ζ(t)→0 together with Φ(t)→π; see (A5). This was indeed observed numerically, as seen below. However, there is also a regime that is not described by (12)–(18), where Φ(t)→π for sufficiently long but finite times, and where ζ(t) stays nonzero; see below for details. This regime is realized when back-reaction parameter ϵ is sufficiently large. As we emphasized, the above analytic derivations do not hold for this case. Eventually, the fact of large back-reaction leads to decaying of the Φ=π state, i.e., Φ=π turns out to be a metastable state.

Lastly, the left-hand side of (22) is just the initial dimensionless energy of ξ; cf. (7) and (11). We call this quantity the initial stored energy, and (22) was obtained for vanishing back-reaction ϵ→0.

## 4. Scenarios of (De-)Stabilization for the Inverted Pendulum

### 4.1. Asymptotic Stability and Stability with Respect to Several Perturbations

**Figure 2:**
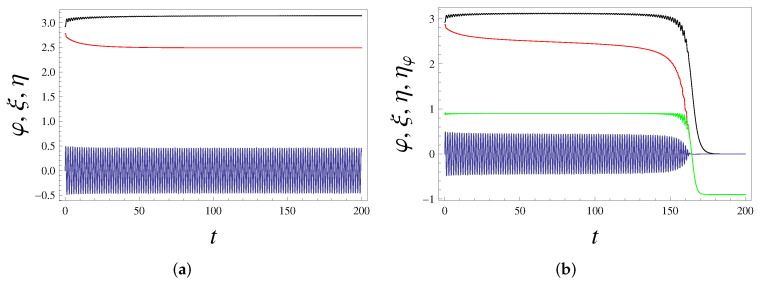
(**a**) Angle variable φ (black, upper curve) and controller ξ (blue, oscillating curve) as the numerical solutions of (6) and (9) versus time *t*. Red curve (in the middle) is the scaled energy $\eta =\frac{E}{\mu +m}$ that includes stored energy $\frac{1}{2}\left[{\dot{\xi }}^{2}+\omega^{2}\xi^{2}\right]$; cf. (8) and (22). Parameters in (6) and (9) are: ϵ=0.8, $\dot{\xi }\left(0\right)=2$ (ξ(0)=0), φ(0)=0.93π ($\dot{\varphi }\left(0\right)=0$), ω=4, γ=3, m=g=l=1. It is seen that φ quickly stabilizes at φ=π, which is normally an unstable state. The stabilization process takes a relatively small amount of energy. After stabilization, φ and ξ decouple; ξ continues oscillating, and scaled energy η is then constant in time. Stability Condition (22) holds (the difference between its LHS and RHS is 0.4375). (**b**) The same parameters as in (**a**), but back-reaction parameter ϵ=0.9 is slightly larger. We also display $\eta_{\varphi }=\frac{E}{\mu +m}-\frac{1}{2}{\dot{\xi }}^{2}-\frac{\omega^{2}}{2}\xi^{2}$ (green curve, third from top), where $\eta_{\varphi }$ is the (scaled) energy related to the angle variable only; cf. (8). There is a rather long (t∼150) period of metastability accompanied by a slow dissipation of energy. After this, stability is lost: φ quickly relaxes to the minimal φ=0 of the potential, and ξ loses all its energy and eventually stops moving (i.e., ξ(t)→0). The whole stored energy dissipates. The physical reason of scenario in (**b**) is that fast oscillations around φ=π do not disappear, i.e., they persist in the metastable state, continuously dissipate energy [cf. (10)], and once the initial energy decreases sufficiently, φ and ξ (relatively) suddenly move to global energy minima $\varphi =\dot{\varphi }=\xi =\dot{\xi }=0$. Energy $\frac{1}{2}{\dot{\xi }}^{2}+\frac{\omega^{2}}{2}\xi^{2}$ stored in ξ decays in time. To confirm this, (**b**) also shows the scaled energy $\zeta_{\varphi }$ related to φ. It stays constant in time for the whole metastability period; see the green curve. A similar scenario happens, when the initial condition φ(0) is out of the attraction basin of φ=π. However, here φ(t) never reaches π.

Figure 2a shows the solution of (6) and (9) when condition (22) holds. State φ=π is indeed asymptotically stabilized, φ(t)→π at least when Φ(0) is sufficiently close to π, i.e., when φ(0) is in the attraction basin of π. This relaxation was accompanied by the energy dissipation. The decaying (dissipating) quantity here is mostly the stored energy $\frac{1}{2gl}\left[{\dot{\xi }}^{2}\left(t\right)+\omega^{2}\xi^{2}\left(t\right)\right]$, i.e., the dimensionless energy related to $E_{\xi }$ in (11). Once φ approaches π sufficiently close, the coupling between ξ and φ is switched off; recall the discussion after (22). This means that the stored energy is not dissipated anymore and stays constant for subsequent times; see Figure 2a. Thus, we confirmed that the originally unstable fixed point of the pendulum can be rendered asymptotically stable without permanent energy costs, but with only transient energy dissipation.

For parameters of Figure 2a, point φ=π is asymptotically stable with a well-defined attraction basin, i.e., it is stable with respect to a single perturbation, which refers to initial state φ(0)=0.93π, $\dot{\varphi }\left(0\right)=0$, and specific initial state $\left(\xi \left(0\right),\dot{\xi }\left(0\right)\right)$ of ξ; see Figure 2a. It is necessary to generalize the notion of asymptotic stability because it is unrealistic to consider only a single perturbation. Let us assume that, after φ(t) relaxed to π, we apply at some random time τ (which is larger than the relaxation time) yet another (second) perturbation φ=π→φ(0)=φ(τ) within the same attraction basin, i.e., φ(0)=0.93 for parameters of Figure 2b. The initial state of ξ is then reset as $\left(\xi \left(\tau \right),\dot{\xi }\left(\tau \right)\right)$. Hence, we now run the dynamics anew with initial states φ(0)=0.93π, $\dot{\varphi }\left(0\right)=0$, ξ(τ) and $\dot{\xi }\left(\tau \right)$.

For the parameters of Figure 2a, φ=π is unstable after the second perturbation. The issue here is that the back-reaction ϵ is sufficiently large; hence, the second perturbation alters the controlling degree of freedom ξ(t) and dries out its stored energy, which already decreased after the first perturbation. If, for parameters of Figure 2a, we decrease ϵ from 0.8 to 0.1, φ becomes stable to many well-separated (in the above sense) perturbations coming at random times. One reason for this is that the stored-energy decrease within one perturbation is smaller. Another reason that numerically clearly differs from the first one is that the motion of ξ becomes more stable with respect to resetting the initial conditions of φ during the perturbation.

### 4.2. Random Noise

Above, we assumed multiple strong and well-separated (in time) perturbation. Another way of implementing multiple perturbations is to include external noise in (9). Let us add to the right-hand side of (9) white Gaussian random noise:

(23) $$\sigma f\left(t\right),\;\langle f\left(t\right)\rangle =0,\;\langle f\left(t\right)f\left(t^{\prime }\right)\rangle =\delta \left(t-t^{\prime }\right),$$

where σ is the noise intensity. The modified (9) then becomes the Langevin equation (for φ(t)). In contrast to strong and well-separated (in time) perturbations, (23) allows for uncorrelated, densely located perturbations that are (most probably) weak if σ is small.

**Figure 3:**
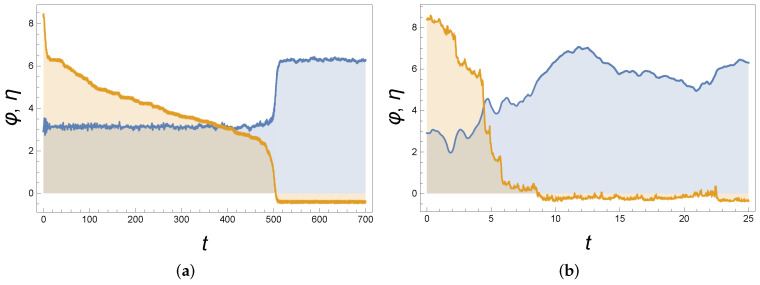
(**a**) Angle φ (blue, piecewise constant curve) as the numerical solution of (6) and (9) versus time *t*, where to the RHS of (9) we added white noise f(t) with intensity σ=0.1; see (23). Here, $\eta =\frac{E}{\mu +m}$ (orange, decaying curve) is the scaled energy; cf. (8). Parameters in (6) and (9) are: ϵ=0.4, $\dot{\xi }\left(0\right)=4$ (ξ(0)=0), φ(0)=0.93π $\left(\dot{\varphi }\left(0\right)=0\right)$, ω=4, γ=3, m=g=l=1. It is seen that ϕ is stabilized around π in a metastable state whose lifetime is ≃500. During this lifetime, energy η slowly decays till it is below some critical value, and then the metastable state suddenly decays. (**b**) The same as in (**a**), but for a stronger noise σ=1.25. Metastable decay is smeared, but still clearly visible.

Figure 3a shows that a weak-random noise monotonously dries out the stored energy; hence, the stability of φ=π is lost after a sufficiently long time. Figure 3b shows that the same scenario hold for stronger noise, though in somewhat blurred form and for a shorter lifetime of the metastable state. For parameters of Figure 3a and σ=0, φ=π is asymptotically stable with respect to 10–11 strong, well-separated perturbations.

This metastability under noise differs from the well-known noise-induced escape from a local energy minimum to a deeper minimum. There, a sufficiently strong random kick brings the system to the attraction basin of the deeper minimum. In contrast, here, the attraction basin is not changed (and no strong kicks are to be waited for). Rather, the stored energy needed for maintaining the stability is drained out and the very local minimal state is destroyed. Hence, what we described here constitutes a new type of noise-induced metastability that deserves further study.

### 4.3. Metastability

For parameters of Figure 2a, if back-reaction ϵ is larger than 0.8, another interesting scenario takes place: the notion of asymptotic stability with respect a single perturbation is lost and replaced by metastability; see Figure 2b.

Now, small oscillations of φ(t) around Φ=π persist and do not decay in time; cf. (12). According to (10), these oscillations slowly drain out the initial stored energy $\frac{1}{2gl}\left[{\dot{\xi }}^{2}\left(0\right)+\omega^{2}\xi^{2}\left(0\right)\right]$, and when it gets sufficiently low, the metastable state Φ=π suddenly decays to the global energy minimum $\varphi =\dot{\varphi }=\xi =\dot{\xi }=0$; see Figure 2b. During this sudden decay, the whole stored energy is dissipated away. In the metastability time window, energy $E_{\varphi }$ related to φ stays constant; see (11) and Figure 2b.

The transition between regimes in Figure 2a,b, i.e., between the truly stable and the metastable state takes place for a critical value of the back-reaction parameter $\epsilon_{c}$. For parameters of Figure 2a,b, we have $\epsilon_{c}\approx 0.86075$. We checked numerically that the lifetime of the metastable state could be very large for ϵ approaching $\epsilon_{c}$ from above. Hence, this time can be arbitrary large for $\epsilon \to \epsilon_{c}+0$.

The transition between regimes in Figure 2a,b is described for the initial fixed conditions of φ: φ(0)=0.93π ($\dot{\varphi }\left(0\right)=0$), i.e., for a fixed attraction basin of the stabilized state. If these initial conditions are changed by rendering φ(0) closer to the stability point π (i.e., the attraction basin is shrunk), then the transition from the stable to metastable regime takes place at a larger value of ϵ or does not take place at all. Likewise, if the attraction basin is enlarged, the transition taken place for smaller ϵ. For example, if φ(0)=0.92π ($\dot{\varphi }\left(0\right)=0$), then the transition takes place at $\epsilon_{c}≃0.6536$, while for φ(0)=0.935π ($\dot{\varphi }\left(0\right)=0$), the solution is stable for all ϵ<1, which is the physical range of ϵ for parameters of Figure 2a,b; cf. (7). The stabilization with the largest attraction basin demands vanishing values of ϵ. In particular, for parameters of Figure 2a, no stabilization of the φ=π state occurs for |φ(0)|<0.64105π ($\dot{\varphi }\left(0\right)=0$), while for |φ(0)|≳0.64105π the stabilization demands ϵ→0.

The metastability in Figure 3a,b is different from that in Figure 2b because, in the latter case, the metastability is due to a strong back-reaction and not permanently acting perturbations.

## 5. Summary and Discussion

The purpose of this work is to understand energy costs of dynamical stabilization (homeostasis): a process that stabilizes an unstable state due to an active controlling process. To this end, one needs plausible models with a well-established history of physical [1,2,3,4,17,18,19,20,21,22,23] and control-theoretic [5,6,7,8,13,14] applications. Here, we studied the inverted (Kapitza’s) pendulum model, where the upper (normally unstable) state is stabilized by a fast motion of a controlling degrees of freedom. Usually, this degree of freedom is replaced by an external field. Here, we modeled it explicitly because we wanted to study an autonomous system with full control of energy and its dissipation. Our main results are summarized as follows.

The unstable state of the pendulum can be asymptotically stabilized with a finite attraction basin and without permanent energy dissipation because the controller–pendulum interaction is automatically switched off once the pendulum is stabilized. There is only a transient dissipation of a small amount of energy related to the stabilization. This regime is reached when both the back-reaction of the pendulum to the controller is sufficiently small and the controller oscillates sufficiently fast, i.e., it has sizeable stored energy.

However, the notion of asymptotic stability was not sufficient: we needed to study stability with respect to multiple perturbations. We implemented two scenarios for multiple perturbations: strong, widely separated in time perturbations, and weak white noise acting on the pendulum. An asymptotically stable state may not be stable with respect to several perturbations. The latter type of stability is achieved only if the back-reaction to the controller is small. In the second scenario, weak white noise led to the noise-driven decay of a metastable state. This decay differed in several ways from the usual noise-driven escape. There is a need for further, more systematic research on this effect.

When the back-reaction was larger, the very notion of asymptotic stability was lost and replaced by metastability. The stabilization was then temporary (metastable), because small oscillations around the stabilized state do not decay. They dry out the stored energy of the controller, and once it is lower than some threshold, the metastable state decays. In this case, a constant dissipation rate was needed for supporting the metastable state.

The energy cost problem was actively studied for the case of adaptation [24]. Here, stability was required with respect to external changes of intensive variables; e.g., temperature and chemical potential [24,25,26,27,28]. Adaptation should be distinguished from the proper dynamical stabilization (homeostasis) [29]. Adaptation is about the stability of intensive variables (e.g., temperature) and relates to structural changes in the system, while homeostasis does not. Hence, adaptation is thermodynamically restricted by the Le Chatelier–Braun principle, whereas homeostasis is not [26,29,30]. Existing approaches to the energy cost of adaptation show that the energy is to be dissipated continuously if an adaptive state is maintained [25,26,27,28]. In that sense, adaptation is similar to proofreading and motor transport, biological processes that are essentially nonequilibrium and demand constant dissipation of energy; see [31] for a review. The question of relating dynamical stabilization and adaptation more directly remains open; so far, the two concepts have been quite separate.

Conceptual tools gained from the physical model should be useful for studying the energy cost of biological examples of dynamical stabilization (homeostasis) [9,10,11,12]. Biological and physiological discussions imply that homeostasis is needed for controlling (and providing advantages for) metabolic processes in organisms [9,10,11,12]. This demands asking about the proper energy costs of the homeostasis itself. Such costs can be substantial, e.g., humming birds (colibri), for which energy saving is crucial, fall at night into a torpor state that is different from normal sleep. In this state several homeostatic mechanisms including internal energy regulation are ceased. Thereby birds are able to save a substantial amount of energy: an increase of ≃60% of the normal usage [32].

Since realistic models of homeostasis are derived from systems biology [9,24,33], they frequently lack a physical form that allows for us to ask questions about energy balance, let alone its dissipation. Nonetheless, some analogies can be drawn and might prove useful in future research. We saw that the energy stored in the controlling degree of freedom can be the main resource of homeostasis. In that respect, it is similar to the energy stored in the living organism, one of the major concepts in biological thermodynamics [34,35,36,37,38,39,40]. It also relates to adaptation energy introduced in physiology [24]; cf. [29] for a critical discussion. The stored energy is phenomenologically employed in dynamic energy budget theory (DEBT) [39,40] and applied for estimating metabolic flows of concrete organisms.

This concept is not yet well-formalized, but some of its qualitative features are known. The stored energy is not the usual free energy, since the latter is present in equilibrium as well. From the viewpoint of a modern thermodynamics, the notion of stored energy resembles energy kept at a certain negative temperature because it is capable of doing work in a cyclic process [41]. For the inverted pendulum, the stored energy is mechanical (not chemical) and relates to an oscillating degree of freedom, but it is also capable of performing cyclic work. The inverted pendulum demonstratess how stored energy relates to stabilizing unstable states.

## Data Availability

Not applicable.

## Appendix A. Derivation of the Effective Potential

Here we explain how to solve Equations (15) and (16), and deduce the effective potential from that solution. Define from (7)

(A1) $$\beta \equiv \frac{\epsilon \;\sin^{2}\Phi }{l}=\frac{\sin^{2}\Phi }{1+\frac{\mu }{m}}<1,$$

and solve (15) and (16) via the Laplace transform as:

(A2) $$\hat{\xi }\left(s\right)=\frac{\left(1-\beta )a(s\right)}{\left(s^{2}+\omega^{2}\right)\left(s+\gamma \right)-\beta s^{3}},\;\hat{\zeta }\left(s\right)=-\frac{\sin\Phi }{l}\;\frac{\left(1-\beta )b(s\right)}{\left(s^{2}+\omega^{2}\right)\left(s+\gamma \right)-\beta s^{3}},$$

(A3) $$a\left(s\right)=\left(s+\gamma \right)\dot{\xi }\left(0\right)+s\left(s+\frac{\gamma }{1-\beta }\right)\xi \left(0\right),\;b\left(s\right)=s\dot{\xi }\left(0\right)-\xi \left(0\right)\frac{\omega^{2}}{1-\beta }$$

where in addition to initial condition (17) we also employed (18).

The inverse Laplacian transform taken from (A2) reads

(A4) $$\xi \left(t\right)=\left[\frac{e^{s_{1}t}a\left(s_{1}\right)}{\left(s_{2}-s_{1}\right)\left(s_{3}-s_{1}\right)}+\frac{e^{s_{2}t}a\left(s_{2}\right)}{\left(s_{1}-s_{2}\right)\left(s_{3}-s_{2}\right)}+\frac{e^{s_{3}t}a\left(s_{3}\right)}{\left(s_{1}-s_{3}\right)\left(s_{2}-s_{3}\right)}\right],$$

(A5) $$\zeta \left(t\right)=-\frac{\sin\Phi }{l}\left[\frac{e^{s_{1}t}b\left(s_{1}\right)}{\left(s_{2}-s_{1}\right)\left(s_{3}-s_{1}\right)}+\frac{e^{s_{2}t}b\left(s_{2}\right)}{\left(s_{1}-s_{2}\right)\left(s_{3}-s_{2}\right)}+\frac{e^{s_{3}t}b\left(s_{3}\right)}{\left(s_{1}-s_{3}\right)\left(s_{2}-s_{3}\right)}\right],$$

where $s_{1}$, $s_{2}$ and $s_{3}$ solve

(A6) $$s^{3}+\frac{\gamma s^{2}}{1-\beta }+\frac{\omega^{2}s}{1-\beta }+\frac{\omega^{2}\gamma }{1-\beta }=\left(s-s_{1}\right)\left(s-s_{2}\right)\left(s-s_{3}\right)=0.$$

Without loss of generality, we take the following parametrization:

(A7) $$s_{1}=-\Gamma ,\;s_{2}=-\tilde{\gamma }+i\tilde{\omega },\;s_{3}=-\tilde{\gamma }-i\tilde{\omega },$$

(A8) $$\Gamma >0,\;\tilde{\gamma }\geq 0.$$

Now, we calculate $\bar{\zeta \left(t\right)\;\ddot{\xi }\left(t\right)}$. Hence, when taking the product $\zeta \left(t\right)\;\ddot{\xi }\left(t\right)$ all oscillating terms with frequency $\tilde{\omega }$ are to be neglected:

(A9) $$\bar{\zeta \left(t\right)\;\ddot{\xi }\left(t\right)}=-\frac{\sin\Phi }{l}\left(\frac{s_{1}^{2}a\left(s_{1}\right)b\left(s_{1}\right)\;e^{-2\Gamma t}}{{\left[{\left(\tilde{\gamma }-\Gamma \right)}^{2}+{\tilde{\omega }}^{2}\right]}^{2}}+\frac{\left(s_{2}^{2}a\left(s_{2}\right)b\left(s_{3}\right)+s_{3}^{2}a\left(s_{3}\right)b\left(s_{2}\right)\right)\;e^{-2\tilde{\gamma }t}}{4{\tilde{\omega }}^{2}\left[{\left(\tilde{\gamma }-\Gamma \right)}^{2}+{\tilde{\omega }}^{2}\right]}\right).$$

If in (A1), β→0 due to μ/m≫1, we can keep in (A6) only the simplest nonzero order putting there β=0. This approximation is supported if slow variable Φ tends to π, i.e., β gets additional smallness. Now, (A6) reads $\left(s+\gamma \right)\left(s^{2}+\omega^{2}\right)=0$, i.e., we obtain via (A7):

(A10) $$\Gamma =\gamma ,\;\tilde{\gamma }=0,\;\tilde{\omega }=\omega .$$

This means that the term $\propto e^{-2\Gamma t}$ in (A9) can be neglected, and we have

(A11) $$\bar{\zeta \left(t\right)\;\ddot{\xi }\left(t\right)}=\frac{\sin\Phi \;[{\dot{\xi }}^{2}\left(0\right)+\omega^{2}\xi^{2}\left(0\right)]}{l}\;\frac{\omega^{2}}{2(\gamma^{2}+\omega^{2})}.$$

Equation (A11) leads us to (19) and (21).
